# Giant bacillary angiomatosis

**DOI:** 10.4102/sajhivmed.v22i1.1257

**Published:** 2021-07-20

**Authors:** Jeremy Nel, Prudence Ive, Carolina Nel

**Affiliations:** 1 Division of Infectious Diseases, Department of Medicine, Faculty of Health Sciences, University of the Witwatersrand, Johannesburg, South Africa; 2 Department of Anatomical Pathology, Faculty of Health Sciences, University of the Witwatersrand, National Health Laboratory Services, Johannesburg, South Africa

**FIGURE 1:**
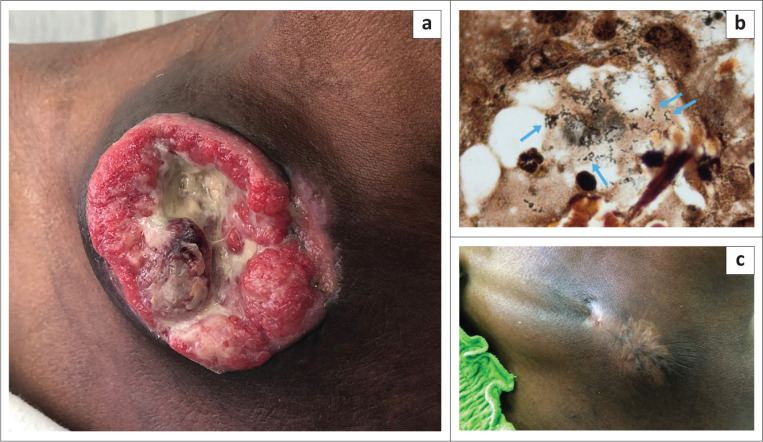
(a) The 20 cm ulcerating bacillary angioma. (b) Warthin-Starry stain highlighting clumps of bacilli in keeping with *Bartonella* species. (c) Resolution of the lesion following 9 weeks of therapy.

A 45-year-old female patient presented with a 2-month history of a progressively enlarging and ulcerating mass on her upper right chest wall, associated with weight loss of 20 kg (Figure 1a). The mass measured 12 cm in diameter and had become gradually more painful as the lesion expanded. The patient was newly diagnosed with HIV-1 infection, with a baseline CD4+ T-cell count of 10 cells/mm^3^ and a viral load of 38 000 copies/mL. Initially a diagnosis of non-Hodgkin’s lymphoma was considered, but a biopsy revealed that the lesion consisted of a proliferation of capillaries lined by plump endothelial cells. A Warthin–Starry stain highlighted bacilli morphologically in keeping with *Bartonella* species (Figure 1b, arrows). An indirect immune fluorescence antibody assay for *Bartonella henselae* immunoglobulin G was strongly positive (> 1:256), and the biopsy sample tested positive for *Bartonella* by polymerase chain reaction, confirming the diagnosis of bacillary angiomatosis. Oral azithromycin therapy resulted in rapid improvement, with abatement of the pain within two days and regression of the lesion to half its original size within two weeks. Antiretroviral treatment was commenced simultaneously. Complete resolution of the lesion was accomplished after nine weeks of therapy, leaving only mild residual scarring (Figure 1c). To the best of our knowledge, this 12-cm lesion is the largest described in the literature to date.

